# Effects of a School-Based Sports Program on Physical Fitness, Physical Activity, and Cardiometabolic Health in Youth With Physical Disabilities: Data From the Sport-2-Stay-Fit Study

**DOI:** 10.3389/fped.2018.00075

**Published:** 2018-03-26

**Authors:** Maremka Zwinkels, Olaf Verschuren, Astrid Balemans, Kristel Lankhorst, Saskia te Velde, Leendert van Gaalen, Janke de Groot, Anne Visser-Meily, Tim Takken

**Affiliations:** ^1^Center of Excellence for Rehabilitation Medicine, Brain Center Rudolf Magnus, University Medical Center Utrecht, Utrecht University and De Hoogstraat Rehabilitation, Utrecht, Netherlands; ^2^Department of Sports, De Hoogstraat Rehabilitation, Utrecht, Netherlands; ^3^Department of Rehabilitation Medicine, Amsterdam Movement Sciences, Amsterdam Public Health, VU University Amsterdam, Amsterdam, Netherlands; ^4^HU University of Applied Sciences Utrecht, Utrecht, Netherlands; ^5^Netherlands Institute for Health Services Research, Utrecht, Netherlands; ^6^Child Development and Exercise Center, University Medical Center Utrecht, Utrecht, Netherlands; ^7^Department of Rehabilitation, Physical Therapy Science and Sports, Brain Center Rudolf Magnus, University Medical Center Utrecht, Utrecht, Netherlands

**Keywords:** sports, physical fitness, physical activity, health promotion, children and adolescents, physical disability, chronic disease

## Abstract

**Objective:**

To investigate the effects of a school-based once-a-week sports program on physical fitness, physical activity, and cardiometabolic health in children and adolescents with a physical disability.

**Methods:**

This controlled clinical trial included 71 children and adolescents from four schools for special education [mean age 13.7 (2.9) years, range 8–19, 55% boys]. Participants had various chronic health conditions including cerebral palsy (37%), other neuromuscular (44%), metabolic (8%), musculoskeletal (7%), and cardiovascular (4%) disorders. Before recruitment and based on the presence of school-based sports, schools were assigned as sport or control group. School-based sports were initiated and provided by motivated experienced physical educators. The sport group (*n* = 31) participated in a once-a-week school-based sports program for 6 months, which included team sports. The control group (*n* = 40) followed the regular curriculum. Anaerobic performance was assessed by the Muscle Power Sprint Test. Secondary outcome measures included aerobic performance, VO_2_ peak, strength, physical activity, blood pressure, arterial stiffness, body composition, and the metabolic profile.

**Results:**

A significant improvement of 16% in favor of the sport group was found for anaerobic performance (*p* = 0.003). In addition, the sport group lost 2.8% more fat mass compared to the control group (*p* = 0.007). No changes were found for aerobic performance, VO_2_ peak, physical activity, blood pressure, arterial stiffness, and the metabolic profile.

**Conclusion:**

Anaerobic performance and fat mass improved following a school-based sports program. These effects are promising for long-term fitness and health promotion, because sports sessions at school eliminate certain barriers for sports participation and adding a once-a-week sports session showed already positive effects for 6 months.

**Clinical Trial Registration:**

This trial was registered with the Dutch Trial Registry (NTR4698).

## Introduction

Daily physical activity is beneficial for all children and adolescents. For those with physical disabilities, similar physical activity recommendations account (1). In addition, exercise interventions have shown that youth with physical disabilities can improve physical fitness levels (2–7) and decrease cardiometabolic risk factors (4–7). Moreover, they have lower levels of physical fitness and participate less in competitive and recreational sports compared to peers who develop typically (3, 8). Since limited physical ability can interfere with being physically active in daily life and consequently affect their health later in life, maintaining sports participation and adequate performance-related fitness levels is especially important in this population (9, 10).

For youth with a physical disability, it seems more difficult to participate in sports and physical activities when compared to typically developing peers (11). In 2011, only 26% of Dutch children and adolescents with a physical disability from schools for special education participate in sports at least once a week compared to 71% in youth without physical disabilities (12). Reasons for this lower sports participation are being physically active is more challenging because of their disability, lack of trained support personnel, transportation problems, lack of acceptance, and no sports clubs in the neighborhood (13–15). Most of these barriers to be physical active can possibly be eliminated when a sports program is provided at school in the immediate after-school hours. This setting offers a familiar environment with supported trainers, acceptance, and no additional transportation except a postponed pickup from school.

In addition, a recent study has proposed the need for school-based initiatives, since their integrative approach is effective and even targets the least active children (16). Recent work in the typically developing population showed positive but inconsistent effects in increasing physical activity with after-school interventions (17, 18). Interventions that lasted more than 12 weeks and that focused solely on increasing physical activity by providing a sports program were most effective (18). According to the exercise principles, a training frequency of at least three sessions a week is recommended to improve cardiorespiratory fitness in youth who are typically developing (19, 20). By contrast, for children and adolescents with cerebral palsy (CP) who are very deconditioned, two sessions a week are also possible to induce or maintain effects (1). On the other hand, from a parents’ perspective, they consider sports participation of once a week to be sufficient (21). Parents need to prioritize the frequency of sports participation with the demands of everyday life (22). In a recent qualitative research, all parents indicated that the intensity or frequency of a sports program to improve fitness levels was not of importance for them. They emphasized the importance of sports as “being active,” having fun, and socialization (21).

For improving fitness levels, there is a discrepancy between exercise recommendations and feasibility in daily life for youth with disabilities and their parents. Providing a once-a-week school-based sports program can relatively easily be implemented and increase children’s level of sports participation. However, it is unknown whether once a week is beneficial for fitness and health purposes in this population. The current study, denoted as the Sport-2-Stay-Fit study, will investigate the effects of a school-based once-a-week sports program on physical fitness, physical activity, and cardiometabolic health in children and adolescents with a physical disability.

## Methods

### Design

The Sport-2-Stay-Fit study is a controlled clinical trial. The study was conducted at four schools for special education in The Netherlands between September 2014 and July 2016. In a previous publication, we described the study design extensively (23). The results of the school-based sports program will be published in two separate papers where this paper focuses on the fitness and health aspects. Both ethics approval and administrative site approvals were granted by the Medical Ethical Committee of UMC Utrecht in The Netherlands (#14-118). This trial was registered with the Dutch Trial Registry (NTR4698).

### Participants

Children and adolescents with a physical disability were recruited *via* four schools for special education in The Netherlands. These schools, dedicated to youth with physical disabilities, have similar learning objectives as regular schools, but the children receive additional attention and support. Children and adolescents were screened by a physical therapist, a physical educator, or a physician for eligibility. Inclusion criteria were (1) a chronic disease or a physical disability; neuromuscular, musculoskeletal, metabolic, or cardiovascular disorder, (2) aged between 6 and 19 years, (3) participation in sports less than twice a week during leisure time in the preceding 3 months or advised to participate in sports by their physical therapist or a physician (4) understands simple instructions, and (5) were expected to be able to perform the physical fitness tests. Exclusion criteria were (1) having a progressive disease, (2) using a powered wheelchair for sport purposes, (3) participation in other research that could possibly influence current results. In addition, all parents and participants from 12 years of age provided informed consent prior to study initiation.

### Procedure

Before recruitment, schools (*n* = 4) were assigned as sport or control site. This was directed by the current presence of school-based sports initiated by motivated physical educators. Schools that already provided (*n* = 1) or intended to provide (*n* = 1) school-based sports in addition to the regular curriculum were assigned sport sites. Otherwise, schools were assigned as control sites (*n* = 2). In case school-based sports had been provided before the start of the study, children and adolescents were only included if they had not participated during the preceding 3 months. Regardless of the group enrolled, all participants followed a high-intensity interval training (HIT) for 8 weeks as an initial start-up for their fitness level and to get familiarized with exercise (23). In this way, all participants knew in what group they would be enrolled: HIT and school-based sports (i.e., sport group) or HIT and control (i.e., control group). The focus of the current study is the school-based sports program, results of the HIT are described elsewhere (24). Outcome measures were assessed at baseline (*T*0), after 8 weeks of HIT (*T*1), and at completion of 6-months intervention (*T*2). Similar outcome measures were evaluated at *T*1 and *T*2 except for physical activity. Because no short-term effect was expected on physical activity following HIT (*T*1) (25, 26), the baseline physical activity (*T*0) was used to analyze the effectiveness. All outcome measures across all schools were assessed by the same trained researcher together with research assistants. The assessors were not blinded for group allocation.

### Intervention

All participants performed HIT for 8 weeks, twice a week for 30 min, containing 8–12 series of 30-s all-out exercises. Detailed information about the training schedule is described elsewhere (23). After 8 weeks, the sport group commenced with the school-based sports program. The program was provided once a week for 45 min by an experienced physical educator at school in addition to the regular physical education schedule. In contrast to the regular physical education where half of the time is spent on skill practice, cooperation, and management (27), sport is about moving more intensively. No instructions were given on exercise intensities, but children and adolescents were encouraged to be physically active, play the game, and have fun (21). The content of the lesson was adapted by the physical educator based on the children’s skills and cognitive level. The sports program included, but was not restricted to, soccer, (wheelchair) basketball, (wheelchair) hockey, and/or easy administered games like playing tag. The presence of the participants was documented every session.

### Outcome Measures

Since intermittent bouts of intense exercise reflect children’s daily activity pattern, the primary outcome was anaerobic performance (28). Outcome measures were tested during school hours, except from physical activity, and were subdivided into four different occasions within 2 weeks: (a) height, weight, anaerobic fitness, and strength, (b) aerobic fitness, (c) blood pressure, arterial stiffness, and body composition, and (d) metabolic profile.

## Physical Fitness

### Anaerobic Fitness

Anaerobic performance was measured with the Muscle Power Sprint Test either while running, walking, or propelling a manual wheelchair as described previously (29, 30). This is an intermittent sprint test consisting of three or six 15-m sprints with a standardized rest of 10 s between sprints. Participants who were ambulatory had to complete six 15-m runs, while wheelchair users completed three 15-m sprints at a maximal pace. Both peak power (PP) and mean power (MP) were calculated from the results of the sprints. To assess agility, time was recorded during a 10 × 5-m sprint test where children or adolescents had to sprint 10 times as fast as possible between two lines of 5 m apart without rest (29).

### Aerobic Fitness

For aerobic fitness, both performance (achieved shuttles) and VO_2_ peak were measured during a 10-m shuttle run/ride test (SRT) (31, 32). The SRT is an incremental exercise test where participants had to adjust their running, walking, or wheelchair propulsion pace to the beep signals until they failed to reach the line twice within one level. The test protocol was selected based on their level of mobility as described previously (23). During the SRT, a calibrated mixing chamber Cortex Metamax 3X (Samcon bvba, Melle, Belgium) was used to measure VO_2_ peak. Metabolic stress test software (Metasoft Studio) was used to measure oxygen uptake (VO_2_), carbon dioxide production (VCO_2_), peak heart rate (HR), and respiratory exchange ratio (RER) = (VCO_2_/VO_2_). Each test lasted until exhaustion. To determine whether a subject reached their maximal effort, two out of the following three criteria had to be achieved: HR ≥ 180 bpm, RER ≥ 1.00 at peak exercise, or subjective signs of intense effort, such as sweating, facial flushing, or a clear unwillingness to continue.

### Strength

Grip strength was measured using a hand-held dynamometer (CITEC, CIT Technics, Haren, The Netherlands). The mean grip strength was calculated out of three attempts with the preferred hand. To assess the explosive strength, either the standing-broad jump or the one-stroke push was performed in those who were ambulatory and propelling a wheelchair, respectively (33). The standing-broad jump referred to the distance jumped with two legs together, while the one-stroke push referred to the distance covered in their wheelchair by one push.

## Physical Activity

The total physical activity was measured objectively using the Activ8 activity monitor (2M Engineering, Valkenswaard, The Netherlands). The system measures acceleration in three planes and is valid to detect six types of activities in persons who are ambulatory: lying, sitting, standing, walking, cycling, and running (34). Participants wore the Activ8 for 7 consecutive days. The device was fixed with Tegaderm™ waterproof skin tape on the ventral side of the upper leg allowing participants to take a shower or a swim. At least two school days with a minimum of 600 min wear time was needed for a representative weekday. For weekend days, at least 1 day of 600 min wear time was required (35). The time spent lying and sitting (sedentary time) and the time spent standing, walking, cycling, and running (active time) were calculated in minutes. Children and adolescents who were manual wheelchair-using (*n* = 9) wore the device as well. However, we omitted the data from the analyses since this device has not yet been validated for wheelchair users.

## Cardiometabolic Health

### Cardiovascular

Both resting blood pressure and arterial stiffness were noninvasively measured with the Arteriograph (Litra BV, Amsterdam, The Netherlands). The measurement was performed in a supine position using an inflatable cuff on the right upper arm. Participants rested supine for 10 min prior to the recording, and they were asked not to move or talk during the test. Arterial stiffness contained two independent values: pulse wave velocity (PWV) and the augmentation index (AIx). The PWV was measured as the speed at which an aortic pulse travels; increased speed indicates stiffer arteries. The AIx provides information on the peripheral resistance of the endothelial vessels; increased index indicates a higher peripheral resistance. To control for differences in sex and age, *Z*-scores of AIx were calculated according to reference values of Hidvégi et al. (36).

### Metabolic

Height and weight were measured to determine body mass index (BMI). A detailed description has been described previously (23). To control for differences in age, *Z*-scores of BMI were calculated according to Dutch reference values (37). For waist and hip circumference, a horizontal measure was taken at the umbilicus and trochanter major, respectively. Fat mass was measured in a supine position with bioelectrical impedance analysis, using the Bodystat Quadscan 4000 (Euromedix, Leuven, Belgium). To determine the metabolic profile, a finger puncture was performed. This was an optional measurement, and consent was asked separately to parents and participants from 12 years of age. During this procedure, blood was drawn through a finger puncture from which the total cholesterol, low-density lipoprotein (LDL), high-density lipoprotein (HDL), fasting glucose, and triglyceride were analyzed. The analyses were performed using a Cholestech LDX analyzer (Mediphos Medical Supplies BV, Renkum, The Netherlands). All participants were instructed not to eat or drink for 3 h prior to this procedure. Before the finger puncture, participants were asked about the fasting period, and if possible, the measurement was postponed. Otherwise, only total cholesterol, LDL, HDL, and triglyceride data were used for analysis (38).

## Data Analysis

A sample size calculation showed that 32 participants per subgroup were needed to detect a 20% difference between groups in anaerobic performance (23). Statistical analysis was performed using SPSS for Windows (version 21.0, SPSS Inc., Chicago, IL, USA) with a statistical significance level of *p* = 0.01 to correct for testing multiple hypotheses. Descriptive statistics were presented as means and standard deviation. To determine the intervention effect, linear regression analyses were performed according to the intention to treat principle. In the linear regression analyses, the outcome measures at *T*2 were the dependent variables, with group allocation and the measured outcome at *T*1 as independent variables. Since participants were not randomly allocated, the outcome measures at *T*1 were included in the analyses to correct for potential baseline differences between groups. In addition, baseline differences in subject characteristics were checked. Subgroup response patterns on age, sex, and mobility level were analyzed and included as confounders in the analyses when they changed the intervention effect. Besides, response patterns of the different schools were analyzed and included as a cluster variable if they changed the intervention effect. Data were graphically checked for normal distribution using residual plots. Variables with non-normally distributed residuals were logarithmically transformed prior to linear regression, after which the results were transformed back, providing a between-group regression coefficient which has to be interpreted as a ratio. The residuals of all variables were normally distributed after logarithmic transformation. Since the dataset was expected to contain incomplete data for some variables, we used multiple imputations to create and analyze 10 imputed datasets. The imputation model included the outcome measures and sex, height, age, weight, and mobility level in the regression model. Regression coefficients (β) and the 95% confidence interval were reported for the regression model. For clinical purposes, the estimated marginal means of both groups, the mean difference, and relative effect (%) were calculated. Linear regression analyses were both performed on the original data and multiple imputation data. Since both models resulted in similar effects, only the multiple imputation models are shown here.

## Results

A total of 138 participants were invited to participate between September 2014 and November 2015 of whom 78 decided to participate. Seventy-one children and adolescents participated in the current study (Table 1). Due to practical reasons, one participant of the sport group was not able to attend the sports program and was therefore assigned to the control group. Prior to the start of the study, sports participation at *T*0 did not differ between the sport [1.1 (1.1) times a week] and control group [0.8 (0.8) times a week] (*p* = 0.285), whereas at *T*2 sports participation differed significantly between the sport [2.1 (1.0) times a week] and control group [0.9 (0.8) times a week] (*p* < 0.001).

**Table 1:** Characteristics of participants.

	Sport (*n* = 31)	Control (*n* = 40)
Age (years), mean (SD)	13.4 (3.0)	14.0 (2.8)
Sex, *n* male (%)	23 (74)	16 (40)
Height (cm), mean (SD)	156.4 (17.5)	157.1 (10.4)
Weight (kg), mean (SD)	54.4 (20.8)	56.9 (18.6)
**Level of mobility, *n* (%)**		
Able to run	22 (71)	15 (38)
Able to walk	6 (19)	19 (47)
Wheelchair user	3 (10)	6 (15)
**Diagnoses, *n* (%)**		
Neuromuscular	22 (71)	35 (87)
–*Cerebral palsy*	10 (32)	16 (40)
–*Spina bifida*	–	5 (12)
–*Psychomotor retardation*	4 (13)	5 (12)
–*Acquired brain injury*	2 (97)	1 (3)
–*Other*	6 (19)	8 (20)
Cardiovascular	2 (6)	1 (3)
Metabolic	4 (13)	2 (5)
Musculoskeletal	3 (10)	2 (5)

**Figure 1:**
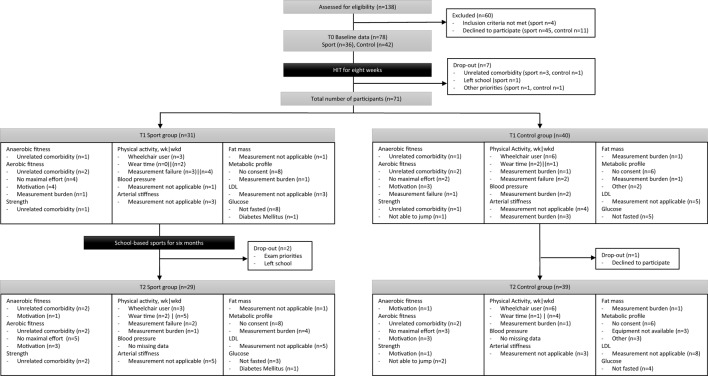
A flowchart from initial inclusion to intervention and reasons for missing data per outcome measure.

Following the school-based sports program, three participants dropped out and some participants did not complete all assessments as illustrated in Figure 1. According to the finger puncture, 74% and 85% of the participants provided consent for the sport and control group, respectively. All measured data at *T*1 and *T*2 are shown for physical fitness and physical activity in Table 2 and for cardiometabolic health in Table 3.

**Table 2:** Mean (SD) of physical fitness and physical activity before (*T*1) and after (*T*2) the school-based sports program.

		*T*1				*T*2			
		*n*	Sport Mean (SD)	*n*	Control Mean (SD)	*n*	Sport Mean (SD)	*n*	Control Mean (SD)
Anaerobic fitness	Anaerobic performance—MP (W) Anaerobic performance—PP (W) Agility (s)	30 30 30	247.9 (188.7) 300.0 (226.5) 24.6 (6.9)	39 39 39	144.0 (107.2) 167.3 (128.3) 28.4 (7.0)	26 26 26	279.2 (208.7) 326.7 (227.3) 25.2 (8.4)	38 38 38	146.3 (109.5) 168.7 (124.7) 28.2 (6.9)

Aerobic fitness^a^	Aerobic performance (shuttles) VO_2_ peak (ml/kg/min) VO_2_ peak (ml/fat free mass/min) HR peak (bpm) RER VE peak (l/min)	21 20 20 20 20 20	13.5 (3.7) 42.6 (8.8) 58.2 (9.7) 194 (11) 1.11 (0.11) 79.6 (35.6)	33 32 32 32 32 32	14.0 (4.2) 34.6 (7.0) 49.9 (9.4) 184 (16) 1.10 (0.09) 69.5 (28.0)	19 19 19 19 19 19	12.6 (3.4) 37.9 (7.2) 51.9 (6.1) 192 (11) 1.11 (0.07) 72.9 (33.7)	31 31 31 31 31 31	13.6 (4.6) 34.3 (8.0) 50.0 (9.9) 185 (14) 1.11 (0.10) 70.2 (27.2)

Strength	Grip strength (*n*) Standing-broad jump (cm)^b^ One-stroke push (m)^c^	30 27 3	153.1 (88) 110.9 (40.6) 7.65 (0.83)	38 31 6	144.7 (57.6) 71.9 (28.5) 6.78 (2.50)	27 24 3	147.8 (84.8) 110.0 (40.3) 6.00 (0.97)	38 30 6	149.2 (56.2) 75.8 (23.5) 6.21 (2.09)

Physical activity^b^	Sedentary time—week (min) Sedentary time—weekend (min) Active time—week (min) Active time—weekend (min)	25 22 25 22	588.8 (90.4) 518.3 (112.0) 271.7 (63.3) 281.7 (86.3)	29 28 29 28	554.1 (101.9) 540.1 (85.1) 259.2 (104.4) 229.1 (80.9)	21 18 21 18	551.1 (79.2) 494.9 (151.8) 302.1 (69.8) 301.7 (117.1)	31 28 31 28	562.0 (85.1) 544.5 (106.1) 282.1 (83.6) 245.9 (90.3)

*MP, mean power; PP, peak power; VO_2_, oxygen uptake; HR, heart rate; RER, respiratory exchange ratio; VE, ventilation*.

*^a^Included only participants who reached maximal effort*.

*^b^Included only participants who were ambulatory*.

*^c^Included only wheelchair users*.

**Table 3:** Mean (SD) of cardiometabolic health before (*T*1) and after (*T*2) the school-based sports program.

			*T*1				*T*2			
		Ref^a^	*n*	Sport Mean (SD)	*n*	Control Mean (SD)	*n*	Sport Mean (SD)	*n*	Control Mean (SD)
Cardiovascular	Systolic blood pressure (mmHg) Diastolic blood pressure (mmHg) AIx (%) AIx (*Z*-score) PWV (m/s)	<130 <80 <15 NA <7	30 30 28 28 28	119.6 (15.3) 64.1 (8.4) 9.82 (9.93) 0.06 (1.25) 5.74 (0.89)	38 38 33 33 33	121.9 (11.4) 67.1 (8.4) 9.58 (8.45) 0.03 (1.22) 6.02 (0.94)	29 29 24 24 25	120.8 (14.6) 65.8 (11.3) 9.50 (10.1) 0.01 (1.16) 5.95 (0.94)	39 39 36 36 36	123.7 (1323) 71.0 (10.3) 12.0 (8.34) 0.49 (1.15) 6.03 (0.81)

Metabolic	BMI (kg/m^2^) BMI (*Z*-score) Waist circumference (cm) Waist–hip ratio Fat mass (%) Total cholesterol (mmol/l) HDL (mmol/l) LDL (mmol/l) Total cholesterol/HDL Triglyceride (mmol/l) Glucose (mmol/l)^b^	<23 NA <85 <0.95 <25 3.0–5.0 >1.0 <3.2 <5.0 0.6–2.2 3.5–5.6	31 31 31 31 30 22 22 19 22 22 13	21.7 (5.4) 1.15 (1.54) 77.3 (14.8) 0.96 (0.05) 26.7 (9.8) 3.88 (0.84) 1.37 (0.48) 1.93 (0.55) 3.07 (1.00) 1.56 (1.00) 5.05 (0.70)	40 40 39 39 39 31 31 26 31 31 26	23.2 (5.2) 1.45 (1.33) 82.4 (15.3) 0.95 (0.08) 32.4 (10.0) 3.85 (0.57) 1.18 (0.29) 2.32 (0.46) 3.43 (0.87) 0.96 (0.52) 4.70 (0.46)	29 29 29 29 28 17 17 12 17 17 13	22.2 (6.1) 1.16 (1.64) 78.4 (16.4) 0.97 (0.06) 25.9 (9.5) 3.78 (0.80) 1.31 (0.46) 2.07 (0.50) 3.14 (1.07) 1.10 (0.72) 4.80 (0.62)	39 39 39 39 38 27 27 19 27 27 23	23.5 (5.1) 1.39 (1.39) 84.3 (16.1) 0.99 (0.07) 32.7 (8.93) 3.99 (0.58) 1.23 (0.30) 2.28 (0.52) 3.43 (0.96) 1.28 (0.94) 5.00 (0.55)

*AIx, augmentation index; PWV, pulse wave velocity; BMI, body mass index; HDL, high-density lipoprotein; LDL, low-density protein*.

*^a^Cutoff reference values of Hidvégi et al. (36), Talma et al. (37), Bodystat Quadscan 4000, and Cholestech LDX software*.

*^b^Included only participants who were fasted*.

Adherence to the sports program was on average 86%, with children and adolescents attending on average 14.4 (4.1) with a range of 5–20 sport sessions. To illustrate, five adolescents attended less than 75% of the program, due to surgery (*n* = 1), other priorities (*n* = 1), and truancy (*n* = 3). No adverse events related to the sports program were reported. Time between measurements was 6.6 (1.3) months with a range of 4.6–10.6 months. The sport group was measured within 2 weeks after finishing the sports program. The huge range between measurements is due to participants of the control group who left school during the study period and agreed to return to finish the assessments on a different occasion.

## Effect of Intervention

### Physical Fitness

**Figure 2:**
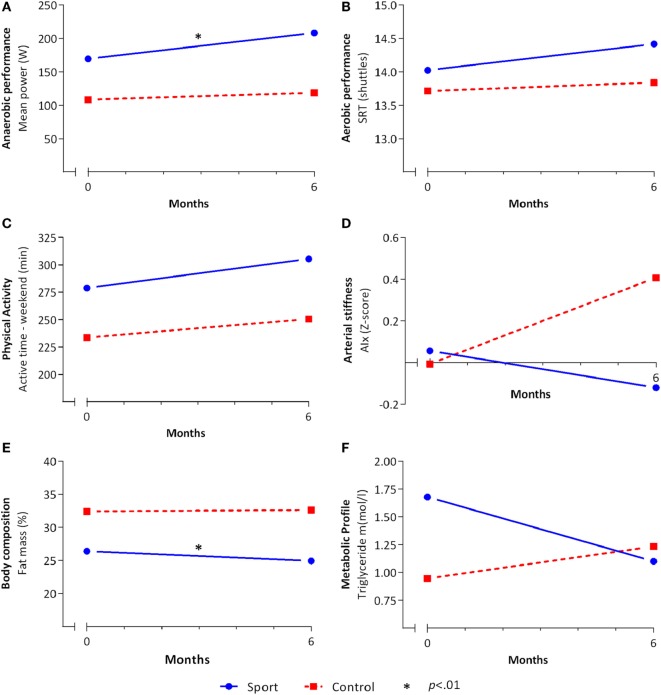
Effects of the school-based once-a-week sports program. The mean values for the sport (solid blue line) and control (dashed red line) group before (*T*1) and after (*T*2) the school-based sports program of **(A)** anaerobic performance, **(B)** aerobic performance, **(C)** physical activity, **(D)** arterial stiffness, **(E)** body composition, and **(F)** metabolic profile on the multiple-imputed model. Linear regression analyses were done on the multiple-imputed model adjusted for *T*1. *Significant (*p* < 0.01) effect in favor of the sport group.

As shown in Figure 2A and Table 4, a significant effect in favor of the sport group was found for anaerobic performance on MP (β = 1.16, IC = 1.05–1.28^1^) and PP [β = 1.15, IC = 1.04–1.27 (see text footnote 1)]. The between-group difference was 23 W (16%) and 25 W (15%) for MP and PP, respectively. No significant effect was observed for agility (Table 4). In addition, no intervention effect was demonstrated for aerobic performance (Figure 2B), VO_2_ peak, and strength (Table 4).

**Table 4:** Results from the linear regression analyses assessing the intervention effect on physical fitness and physical activity.

		Linear regression^a^		Estimated marginal means Differences between groups			
			
		β	95% CI	Sport	Control	MD	%
Anaerobic fitness	Anaerobic performance—MP (W)^b^ Anaerobic performance—PP (W)^b^ Agility (s)^b^	1.16 1.15 1.02	1.05–1.28* 1.04–1.27* 0.96–1.07	164.8 192.3 25.8	142.2 167.1 25.4	22.6 25.2 0.41	16 15 1.6

Aerobic fitness	Aerobic performance (shuttles) VO_2_peak (ml/kg/min) VO_2_peak (ml/fat free mass/min)	0.31 −1.55 −3.00	−1.19–1.81 −4.66–1.56 −7.92–1.92	14.3 35.5 49.5	14.0 37.0 52.5	0.31 −1.55 −3.00	2.2 −4.2 −5.7

Functional strength	Grip strength (*n*) Standing-broad jump (cm)^c^ One-stroke push (m)^d^	−7.72 −0.12 −0.80	−17.97–2.53 −7.31–7.07 −2.86–1.25	147.6 96.8 5.61	155.3 94.0 6.41	−7.72 −0.12 −0.80	−5.0 −0.1 −13

Physical activity^c^	Sedentary time—week (min) Sedentary time—weekend (min) Active time—week (min) Active time—weekend (min)	−26.7 −40.6 8.0 33.7	−66.8–13.4 −119.0–37.8 −31.4–47.4 −40.1–107.4	544.8 497.8 294.7 291.5	571.6 538.4 286.6 257.8	−26.7 −40.6 8.0 33.7	−4.7 −7.5 2.8 13

*p < 0.01.

*CI, confidence interval; MD, mean difference; MP, mean power; PP, peak power; VO_2_, oxygen uptake*.

*^a^Multiple imputation models adjusted for baseline values. The intervention effect was not substantially confounded by age, sex, level of mobility, or school*.

*^b^Regression coefficients should be interpreted as a ratio as it was logarithmically transformed*.

*^c^Included only participants who are ambulatory*.

*^d^Included only wheelchair users*.

### Physical Activity

At baseline, no seasonal differences were found among participants measured in the autumn, winter, and spring. Sports participation was increased during the school-based sports program in the sport group with 1.2 (0.9) times a week compared to 0.1 (0.9) times a week in the control group (*p* < 0.001). Between-group differences showed no intervention effect on physical activity. Sedentary and active time during both week and weekend days showed no between-group difference following the school-based sports program (Table 4; Figure 2C).

### Cardiometabolic Health

No effects were found for blood pressure and arterial stiffness (Table 5). Although not statistically significant, a change of 0.53 *Z*-score on AIx might be clinically relevant (Figure 2D). A significant effect in favor of the sport group was demonstrated for fat mass (β = −2.78, IC = −4.78 to −0.78). The sport group lost 2.8% more fat mass compared to the control group (Figure 2E). No effects were observed for BMI, waist–hip ratio, and metabolic profile. For triglyceride, a small but nonsignificant effect was found [β = 0.64, IC = 0.43–0.94 (see text footnote 1)] with a between-group change ratio of −0.44 mmol/l in favor of the sport group (Figure 2F).

**Table 5:** Results from the linear regression analyses assessing the intervention effect on cardiometabolic health.

		Linear regression^a^		Estimated marginal means Differences between groups			
			
		β	95% CI	Sport	Control	MD	%
Cardiovascular	Systolic blood pressure (mmHg) Diastolic blood pressure (mmHg) AIx (%) AIx (*Z*-score) PWV (m/s)	−0.46 −2.57 −3.02 −0.53 0.20	−5.50–4.59 −7.24–2.10 −7.35–1.31 −1.10–0.04− 0.15–0.54	122.5 67.2 8.93 −0.08 6.18	123.0 70.0 11.95 0.45 5.98	−0.46 −2.57 −3.02 −0.53 0.20	−0.4 −3.7 −25 −117 3.3

Metabolic	BMI (kg/m^2^) BMI (*Z*-score) Waist circumference (cm) Waist–hip ratio Fat mass (%) Total cholesterol (mmol/l) HDL (mmol/l) LDL (mmol/l) Total cholesterol/HDL Triglyceride (mmol/l)^b^ Glucose (mmol/l)^c^	0.05 −0.06 −1.13 −0.02 −2.78 −0.35 −0.07 −0.05 0.02 0.64 −0.31	−0.64–0.90 −0.25–0.14 −3.41–1.15 −0.04–0.00 −4.78 to −0.78* −0.80–0.10 −0.29–0.15 −0.37–0.28 −0.44–0.47 0.43–0.94 −0.68–0.07	22.8 1.23 80.8 0.97 27.7 3.67 1.21 2.08 3.31 0.78 4.80	22.8 1.28 82.0 0.99 30.5 4.02 1.28 2.13 3.29 1.21 5.11	−0.05 −0.06 −1.13 −0.02 −2.78 −0.35 −0.07 −0.05 0.02 −0.44 −0.31	−0.2 −4.3 −1.4 −1.9 −9.1 −8.8 −5.6 −2.2 0.5 −36 −6.0

*p < 0.01.

*CI, confidence interval; MD, mean difference; AIx, augmentation index; PWV, pulse wave velocity; BMI, body mass index; HDL, high-density lipoprotein; LDL, low-density protein*.

*^a^Multiple imputation models adjusted for baseline values. The intervention effect was not substantially confounded by age, sex, level of mobility, or school*.

*^b^Regression coefficients should be interpreted as a ratio as it was logarithmically transformed*.

*^c^Included only participants who were fasted*.

## Discussion

The aim of this study was to evaluate the effects of a school-based once-a-week sports program on physical fitness, physical activity, and cardiometabolic health in youth with physical disabilities. For all participants, both able to walk/run or propel a manual wheelchair, a school-based sports program is feasible and can be performed safely. Despite the heterogeneity of the group, increasing the level of sports participation once a week for 45 min showed already positive effects after 6 months. We found effects in favor of the sport group in both anaerobic performance and fat mass.

The school-based sports program resulted in a positive within-group difference of 23 W (16%) in anaerobic performance. This absolute increase is comparable with the 20 W (38%) improvement after 8 months of exercise training in children and adolescents with CP (39). The higher relative difference, 16% in the current study versus 38%, can be explained by the lower baseline values in the study by Verschuren et al. (39) Moreover, 8 weeks of HIT prior to the school-based sports program resulted already in an increase in anaerobic performance in both sport and control groups of 11% (24). Hence, independent of baseline values, once-a-week sports participation improves anaerobic performance even further. Another remarkable finding is that the control group maintained its gains on anaerobic performance following the regular curriculum of 6 months. It is unknown which factors contribute to this sustainability, but probably youth with physical disabilities are more active in daily life compared to several years ago. A recent cross-sectional study showed that anaerobic performance increased in youth with CP between 2004 and 2014 (40). What we can conclude thought is that children and adolescents with physical disabilities improve anaerobic performance with an extra sports session a week, even after a training period.

The sports program resulted in a positive effect on fat mass, but found no differences in BMI, while other studies reported positive effects on BMI after a school-based intervention program (41, 42). Both fat mass and BMI are generally known to identify adiposity, although BMI fails to distinguish between lean and fat mass and lacks in sensitivity (43, 44). This might explain why BMI remained unchanged in the current study. Possibly, a small shift from fat to lean mass has occurred in the sport group, while weight and consequently BMI remained unchanged. Besides BMI, also no changes were found in waist circumference and waist-to-hip ratio, while other studies showed significant changes in adolescents with CP following an exercise program (4, 6). Possibly, if participants continue with sports participation, or exercise more frequently, in the longer term, these nonsignificant differences in health will diverge positively compared to individuals who do not exercise regularly (45, 46).

The current school-based sports program was performed once a week. Possibly, the frequency of once a week could explain why we found no significant effects on most of the outcome measures. However, once a week reflects daily life, since there is a discrepancy between the requirements from exercise physiology perspectives and the feasibility or the priority of sports participation. Although participants did not train following exercise guidelines, increasing sports participation with once a week improved anaerobic performance and fat mass. Consequently, being active is always better than being inactive (47) and is the starting point for an active and healthy adulthood (48). The current study demonstrated that sports participation of only once a week already shows positive effects after 6 months and tend to induce more effect over a prolonged period. Beside sports participation, daily physical activity also consists of playing outside and active transportation. Compared to typically developing peers, the current population is also less active in these domains of physical activity (12). Families of youth with a physical disability should therefore be encouraged to perform an additional activity in the week or weekends to optimally profit from the benefits of physical activity.

The current study examined the effects of group exercise on various outcome measures. Although we were interested in the group effects, reasons for participation may vary across individuals (21). For example, children and adolescents want to keep up with friends in playing soccer, lose weight, make friends, or just have fun. In the current study, we did not measure these reasons. To establish greater and clinical relevant effects, future research should tailor outcome measures on individual needs. For example, school-based physical activity program targeted at overweight children reduces BMI and blood pressure to a greater extent compared to the general population (41, 42). Moreover, the current school-based sports program did not lead to significant changes in daily physical activity. This probably needs an intervention with a behavioral component, which we did not include. However, earlier research with a behavioral component showed also no effects on physical activity in youth with CP (25, 26). A recent review showed that both parental involvement by education or homework tasks and the inclusion of activities conducted after school time induce greater effects on physical activity and body composition (41).

Several limitations should be taken into account. Firstly, the current study is not controlled by a randomly assigned group. This resulted in very dissimilar groups. Although we corrected for group differences at *T*1, it is difficult to attribute the improvement of anaerobic performance to the sports program only. A second limitation of this study is the composition of the study population comprising a large age range and a variety of diagnoses. More boys and youth who are able to run were included in the sport group compared to the control group. For this reason, the results should be interpreted more carefully. Thirdly, our results cover only the Dutch population of youth with physical disabilities. In The Netherlands, these children with special needs are often assigned to schools for special education, while in other countries, these children follow inclusive education. Therefore, the practical implication of school-based sports programs at schools for special education may be different in other countries.

In conclusion, a school-based once-a-week sports program improved anaerobic performance and fat mass after 6 months in youth with physical disabilities. No intervention effects were found for aerobic performance, VO_2_ peak, strength, physical activity, blood pressure, arterial stiffness, and the metabolic profile. These effects are promising for long-term fitness and health promotion, because in the current study, barriers for sports participation were eliminated by providing sports at school, and only a training volume of once a week was added. Future research on school-based sports programs in this population should tailor outcome measures on individual needs and involve parents to induce greater and clinical relevant effects.

## Ethics Statement

This study was carried out in children and adolescents with a chronic disease or a physical disability in accordance with the recommendations of Good Clinical Practice with written informed consent from all subjects. All parents and participants from 12 years of age gave written informed consent in accordance with the Declaration of Helsinki. The protocol was approved by the Medical Ethical Committee of UMC Utrecht in The Netherlands (#14-118).

## Author Contributions

MZ and KL contributed to the design of the study and collection of data. MZ, AB, SV, and LG helped to analyze the data. OV, JG, AV, and TT conceived of the study, participated in its design and coordination. MZ wrote the manuscript with input from all authors, who read and approved the final manuscript.

## Conflict of Interest Statement

The authors declare that the research was conducted in the absence of any commercial or financial relationships that could be construed as a potential conflict of interest.

## Footnotes

^1^Regression coefficients should be interpreted as a ratio as it was logarithmically transformed.
